# Comparison between using inlet circular jet and elliptic jet in combustion chamber by using twin jet flow propane and methanol

**DOI:** 10.1038/s41598-024-58000-2

**Published:** 2024-04-05

**Authors:** Mohamed M. S. Yaseen, Ahmed A. Attia, M. W. El-Dosouky, Maher Gamil Hegazy, Ismail M. M. Elsemary

**Affiliations:** 1 Combustion and Energy Technology Lab Mechanical Engineering Department, Faculty of Engineering at Shoubra, Benha University, Banha, Egypt; 2 Mechanical Engineering Department, Higher Technological Institute, Tenth of Ramadan City, Al-Sharqia Egypt; 3 Department of Mechanical Engineering, College of Engineering, Northern Border University, 91431 Arar, Saudi Arabia

**Keywords:** Combustion, Efficiency, Heat release rate, Equivalent ratios, Engineering, Mechanical engineering

## Abstract

The current work aimed to improve the combustion behavior of a non-premixed twin-jet inlet. The effect of fuel and air inlet shape under different velocities was studied using ANSYS as the process takes place in species transport and finite rate/eddy dissipation, and the flow is considered to be turbulent. Two different shapes (circular–circular and circular–elliptic inlet jets) were investigated, and the results show that the behavior and intensity of the fire are affected by variations in the speed and, geometry of the inlet which affects temperature, heat release rate, combustion efficiency, and equivalent ratios. The optimum air/fuel velocities were found to be 2.5/1.5 with circular–circular inlet jets.

## Introduction

The development of efficient design of combustors needs to increase in cost of experimental tests and the efforts. A turbulent combustion model with detailed chemical kinetics is needed for accurate predictions of flame stability and pollutant emissions. So that, it is required to develop more efficient, reliable, and robust numerical models. The mixing in twin jets has a wide range in high-speed propulsion systems to reduce noise, thrust vectoring control, etc. Twin jet also enhances combustion efficiency, enhance combustion stability, and significantly reduce emissions. In the early days, mostly circular twin jets were used in manufacturing for easy incorporation with gas turbine engines. The need for non-circular twin jets arose as the necessity for faster large-scale and small-scale mixing increased the spreading rate and better maneuverability. This literature study dives into a variety of studies conducted over several decades to investigate various features of the combustion system, such as combustion stability and twin jet configuration. Moradi et al.^1^ studied the shape effect of cavity flame-holder and found that the trapezoidal cavity is more efficient than other shapes in the preservation of the ignition zone within the cavity. Verma et al.^2^ Used two RANS-based turbulence models, RNG k–ε and k–ω SST turbulence models are used to model turbulence, and the Discrete Ordinate (DO) radiation model is used to model radiation heat transfer between different surfaces in the room and found that the predicted comfort temperature and operative temperature have similar profiles irrespective of the turbulence models. Maele et al.^3^ utilized and compared the RANS-based turbulence models, namely standard k–Ɛ, realizable k–Ɛ, and RNG k–Ɛ turbulence models to the swirling flames in the Sydney burner. It was noticed that the realizable k–Ɛ model yielded a slightly better flow field. The comparison between the standard k–Ɛ model, the modified k–Ɛ model, and the RMS model is carried out by Frassoldati et al.^4^ and they found that the modified k–Ɛ gives better predictions for fuel jet dynamics than RSM. Dally et al.^5^ modified the value of a constant in the dissipation transport equation of the standard k–Ɛ model and found that the modified k–Ɛ gives better results than the Reynolds stress model RMS for bluff body flame.

## Review

To accomplish outstanding of methanol for pollution emissions of nitrogen oxides and carbon monoxide Jing^6^ it was found that blade angel inclination with an equivalent ratio in the range of 1–1.25 is the best performance in the emission of No_x_ and Coopered with other combustion of swirling blade. In the early days, mostly circular twin ejectors were used because of the ease of manufacturing the need for non-circular twin jets arose as a necessity for fast large scale. Muthuram^7^ directing insight about various flow characteristics such as jet flow structure and its development, RANS modeling of premixed turbulent combustion, Fiorina et al.^8^ utilized the tabular chemistry acquired from the premixed flame let and the supposed probability density function (PDF) technique. They enquired about a high-velocity variation of flame with a thickened-wrinkled structure and suggested a scalar dissipation rate established on an appraisal of the conjugation between micro-mixing and flame wrinkling.

Wei et al.^9^ explores using a jet flow from an ignition chamber to improve ignition and flame spread in lean methanol combustion where the experiments show a smaller channel between chambers and leaner fuel mixtures increase jet flow speed, but lower peak pressure due to slower burning. The study suggests a specific fuel-to-air ratio for stable combustion in lean premixed methanol. Fan et al.^10^ improved a rotary engine's combustion by using a mix of methanol and gasoline with Turbulent Jet Ignition (TJI). Test bench and computer model to analyze how pre-chamber size and jet orifice design affect flame propagation was designed. Finding a balance between ignition strength and flame timing, the study suggests optimal values for these design features. The research also revealed a trade-off between lower CO emissions from better combustion and higher NO emissions at peak cylinder pressure.

Bray et al.^11^ employed simplified chemical kinetic processes in turbulent premixed flame models with significant Damköhler numbers for hydrogen-air and methane-air systems. The PDF of two independent scalar variables was utilized. Richardson et al.^12^ enquired about the mixing time scale in 3-D DNS with abbreviated CH_4_-air chemistry in the narrow reaction zones regime of turbulent Bunsen flames. They evidenced that the mixing rates are impacted by the steep slopes accomplished by flame structures with high Damköhler numbers (7–11) furnish a detailed discussion of chemical kinetics reduction approaches with an emphasis on the combustion process.

Tang et al.^13^ formulated a 3-D CFD model with an incorporated skeletal response mechanism for premixed H_2_-air combustion in a thermo-photovoltaic system's micro combustor. The radiation wall of the new combustor design indicated a greater peak mean temperature ascribable heat transfer enhancement, which was more noticeable at higher plate numbers. The thickened flame TF model is among the efficient ways for modeling the flame front in turbulent premixed combustion. To mimic pollutant production, ignition, and extinction, multistep reaction processes become progressively significant.

Gou et al.^14^ demonstrated incomplete combustion in either laminar or turbulent settings for the coupling of the TF model with multistep reaction mechanisms and concluded that the modified dynamic thickening flame sensor is appropriate for multistep reaction. Natural gas fuel had much poorer combustion efficiency and greater combustor pressures for relighting than the other fuels. The poor performance of natural gas is demonstrated to be caused by the chemical stability of the methane molecule, demonstrating that the utilization of natural gas fuel consequences in importantly lower combustion efficiency at severe operating conditions, peakier values of combustor pressure (which happened at lower flight altitude) for altitude ignition and blowout, and a greater proclivity for combustion instability than either ASTM A-l or propane fuel.

Anazadehsayed et al.^15^ investigated the influences of the micro air jet on the mixing of the sonic transverse hydrogen through micro-jets subjected to a supersonic crossflow and found that the mixing performance of the fuel jet is increased by more than 200% when the micro air jet is injected. Consequently, an enhanced mixing zone occurs downstream of the injection slots which leads to flame-holding.

## Problem definition

While methanol is attractive as a clean fuel due to its very low emissions of nitrogen oxides (NOx) and carbon monoxide (CO), its widespread use in industry faces challenges. One of the biggest hurdles is its lower energy density compared to gasoline. This is because methanol requires significantly more energy to vaporize (latent heat of vaporization is 3.7 times higher) and it also packs less energy per unit volume (low heating value)^16^.

**Figure 1:**
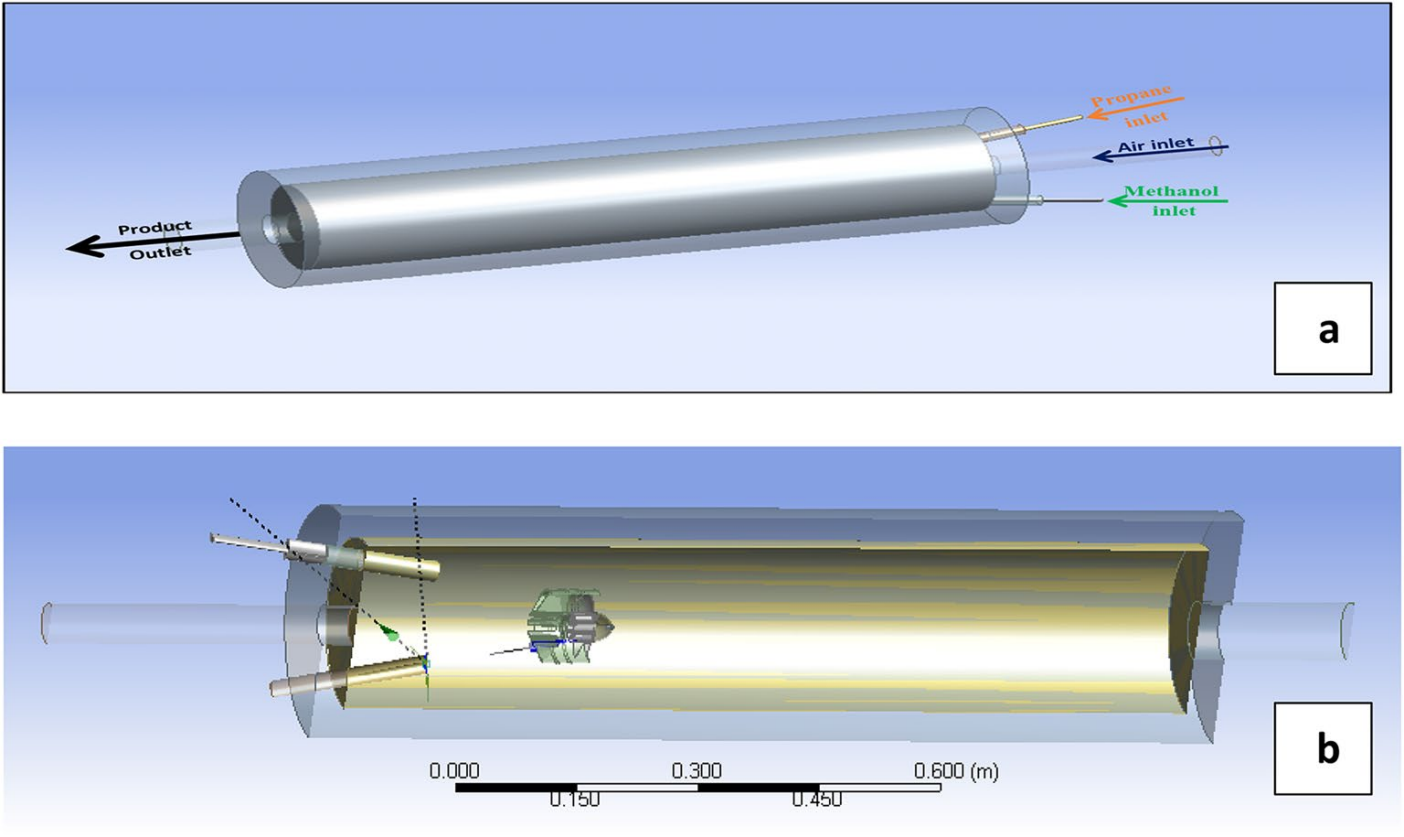
Combustion chamber; (**a**) inlet–outlet, (**b**) cross-section.

To overcome this problem, the present work is concerned with the combustion of non-premixed two types of fuel (twin jets of methanol and propane) injected through two inclined jets with air-jet pipe in a cylindrical combustion chamber. The resulting combustion is then mixed by centrifugal fane which generates turbulent flow. The schematic diagram of the physical model is shown in Fig. 1 and the dimensions of combustion chamber and jets are available in Table 1.

**Table 1:** Combustion chamber and jets dimensions.

Dimension	Value
D1 inside diameter of the combustion chamber	0.22 m
D air pipe	0.05 m
Djet propane	0.0258 m
Djet methanol	0.02589 m
Elliptic jet dimensions	RMN1 = 0.0072065 M RMN3 = 0.0072065 M RMX2 = 0.012945 M
L_Length of combustion chamber_	110 cm
The outer diameter of the combustion chamber	0.30 m

By making this modeling using ANSYS while controlling the data we can deduce that many parameters investigate the combustion performance, behavior, and shape of fire like velocities and geometry of inlet jets which can affect the properties and mass flow rate of combustion firing, twin jets is different from one jet, this work is considered to be optimal for the environment by using clean fuels which is improved. It consists of a combustion chamber consist of an outer diameter of 30 mm, an inner diameter of 22 mm, and a length of 110 mm with an inlet which divided into one pipe of air and twin jets for gases which optically by centrifugal fan with blade inclination 60° * 60° which tabulate the mixture.

### Computational domain and grid

The mesh used for the propellant gas is the face mesh with a 1 mm size, and for the combustion chamber is 5 mm, other bodies are automatically generated. The number of nodes is 44,225 and the number of elements is 218,157.

### The species model

The species were modeled using the model developed by Westbrook and Dryer^17^. This basic model has a double chemical reaction and species.

### Mathematical modeling

#### Turbulence model

The standard two-equation, *k–ε* turbulence model with standard values was used for this study. The *k–ε* turbulence model consists of the turbulent kinetic energy and dissipation equations given below:

1 $$\frac{\partial k}{{\partial t}} + \nabla .\left( {\rho \mathop{u}\limits^{\rightharpoonup} k} \right) = \nabla .\left\{ {\left[ {\mu_{lam} + \frac{{\rho \upsilon_{t} }}{{\sigma_{k} }}} \right]\nabla k} \right\} + \rho \upsilon_{t} G - \rho \varepsilon$$

2 $$\frac{\partial \varepsilon }{{\partial t}} + \nabla .\left( {\rho \mathop{u}\limits^{\rightharpoonup} \varepsilon } \right) = \nabla .\left\{ {\left[ {\mu_{lam} + \frac{{\rho \upsilon_{t} }}{{\sigma_{\varepsilon } }}} \right]\nabla \varepsilon } \right\} + C_{1\varepsilon } \rho \upsilon_{t} G\frac{{\varepsilon^{2} }}{k} - C_{2\varepsilon } \frac{{\varepsilon^{2} }}{k}$$

where *G* represents the turbulent generation rate which is equal to

3 $$G = 2\left\{ {\left[ {\frac{\partial u}{{\partial x}}} \right]^{2} + \left[ {\frac{\partial v}{{\partial y}}} \right]^{2} + \left[ {\frac{\partial w}{{\partial z}}} \right]^{2} } \right\} + \left( {\frac{\partial u}{{\partial y}} + \frac{\partial v}{{\partial x}}} \right)^{2} + \left( {\frac{\partial u}{{\partial z}} + \frac{\partial w}{{\partial x}}} \right)^{2} + \left( {\frac{\partial w}{{\partial y}} + \frac{\partial v}{{\partial z}}} \right)^{2}$$

In the execution of this model the Kolmogorov–Prandtl expression for the kinematic turbulent viscosity, ν_t_ is used, and it is given by:

4 $$\upsilon_{t} = C_{\mu } \frac{{\varepsilon^{2} }}{k}$$

In the equations above Cμ, σk, σϵ, C1μ, and C2μ are all taken to be constants and are given their usual standard values of 0.09, 1.0, 1.3, 1.44, and 1.92 respectively.

The species were modeled using the model based on the work published by Westbrook and Dryer^17^. This simplified model consists of one chemical reaction and 5 species. The mixing and transport of chemical species can be modeled using Fluent-Ansys by solving the conservation equations that describe convection, diffusion, and reaction for each element species. The simultaneous chemical reaction can be modeled here as a volumetric reaction.

The mass fraction of each species, Yi can be obtained by solving the conservation equation for the ith species that can be written as:

5 $$\frac{\partial }{{\partial {\text{t}}}}\left( {\rho Y_{i} } \right) + \nabla .\left( {\rho \mathop{v}\limits^{\rightharpoonup} \upsilon Y_{i} } \right) = - \nabla \mathop{J}\limits^{\rightharpoonup} _{i} + R_{i} + S_{i}$$

where *R*_*i*_ = the net production rate of species *i* by chemical reaction and *S*_*i*_ = the rate of creation by addition from the dispersed phase and any other sources.

Mass diffusion in laminar flows.

In laminar flows, the diffusion flux $\mathop{J}\limits^{\rightharpoonup} _{i}$ of species i in Eq. (1), which results from concentration and temperature gradients can be expressed as:

6 $$\mathop{J}\limits^{\rightharpoonup} _{i} = \rho D_{t,m} \nabla Y_{i} - D_{T,j} \frac{ \nabla T}{T}$$

where *D*_*i,m*_, *D*_*T,i*_ are the mass and the thermal (Soret) diffusion coefficients respectively.

#### Mass diffusion in turbulent flows

The mass diffusion in turbulent flow takes the form:

7 $$\mathop{J}\limits^{\rightharpoonup} _{i} = - \left( {\rho D_{t,m} + \frac{{\mu_{t} }}{{SC_{t} }}} \right)\nabla Y_{i} - D_{T,i} \frac{\nabla T}{T}$$

where Sc_t_ = the turbulent Schmidt number (Sc_t_ = μ_t_/ρD_t_), μ_t_ = the turbulent viscosity. D_t_ = the turbulent diffusivity.

In many multicomponent mixing flows, the transport enthalpy due to diffusion of species is given by: $\nabla . \left[ {\sum\nolimits_{i = 1}^{n} {h_{i} \mathop{J}\limits^{\rightharpoonup} _{i} } } \right]$ which affects significantly when Lewis's number is far from unity.

8 $$Le_{i } = \frac{K}{{\rho C_{p} D_{i,m} }}$$

The reaction rate that appears as a source term in Eq. (1) is computed in Fluent-Ansys by one of the three models, the first is the laminar finite rate model, the second is the eddy dissipation model and the third one is the eddy dissipation concept. Detailed descriptions of these models.

As recommended by Jing et al.^6^, the finite-rate/eddy dissipation model was used to simulate the turbulence-chemistry interaction (gaseous combustion). In essence, the net rate of response is the lowest of the following. The kinetic rate of chemical synthesis or depletion. The rate at which reactant eddies dissipate. The rate at which product eddies dissipate.

The Boundary Conditions used in our model are as follows. The inlet conditions of Air/fuel, Methanol/fuel, and Propane/fuel are tabulated in Table 2. Also, the species mass fraction was determined according to the studied equivalence ratio.

**Table 2:** Inlet conditions of the combustion chamber.

Inlet	Inlet velocity of the air pipe (m/s)	Turbulence intensity (%)	Turbulence length scale (m)	Inlet temperature (k)
Air/fuel	12, 6, 5, 2.5	3.6	0.003	298
Methanol/fuel	2.22,1.2, 1.5, 2	3.6	0.003	298
Propane/fuel	2.22, 2, 1.2, 1.5	3.6	0.003	298

Combustion gases outlet gauge pressure is zero with a backflow temperature of 923 K. Outlet species mass fractions depend on the equivalence ratio. Wall boundary condition has no-slip adiabatic conditions.

### Method of solution

#### Stability and convergence

It is difficult to obtain a convergent solution for reacting flow because of the strong impact of the chemical reaction on the basic flow pattern and the strong coupling between mass and momentum equations and species transport equations. Also, the large heat release from the reaction causes high-density change and a large acceleration in the flow. These coupling issues are solved by using a two-step solution technique. When the reaction rate kinetics is more rapid than the rate of convection and diffusion the solution of species transport becomes more difficult and such a system is termed as “stiff” and the coupled solver is recommended for laminar flow and eddy dissipation concept is recommended for turbulent flow.

To reach a stable converged solution in a reacting flow two-step process can be a practical solution. This can be achieved by solving the governing equations with disabling the reaction i.e. (cold/non-reacting) flow. After reaching the basic flow pattern the reaction can be enabled. Practically, to initiate the spontaneous ignition for fuel/air mixture, the temperature of the mixture should exceed the activation energy threshold requirements. The same issue is made in the current simulation. A finite rate eddy dissipation model for chemistry-turbulence interaction is used. The initial spark was supplied by patching a high-temperature field (1000 K) into the computational domain of combustion. It is noted that the initial patch does not affect the final steady-state solution.

The gradient least square cell-based method was applied for spatial discretization schemes and the second-order upwind scheme was employed for density, momentum, and modified turbulent viscosities. Under relaxation factor of 0.8 was used for species, energy, and density while a value of 0.6 was applied for momentum, turbulent kinetic energy, turbulent kinetic dissipation rate, and turbulent viscosity.

#### Model validation

**Figure 2:**
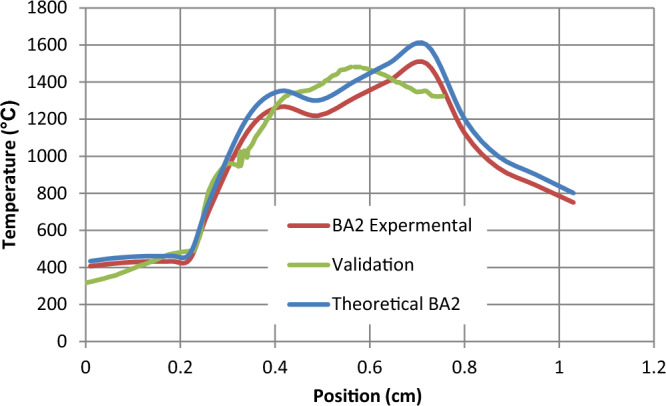
Comparison between the theoretical and experiment of Jing et al.^6^ work with my validation.

**Figure 3:**
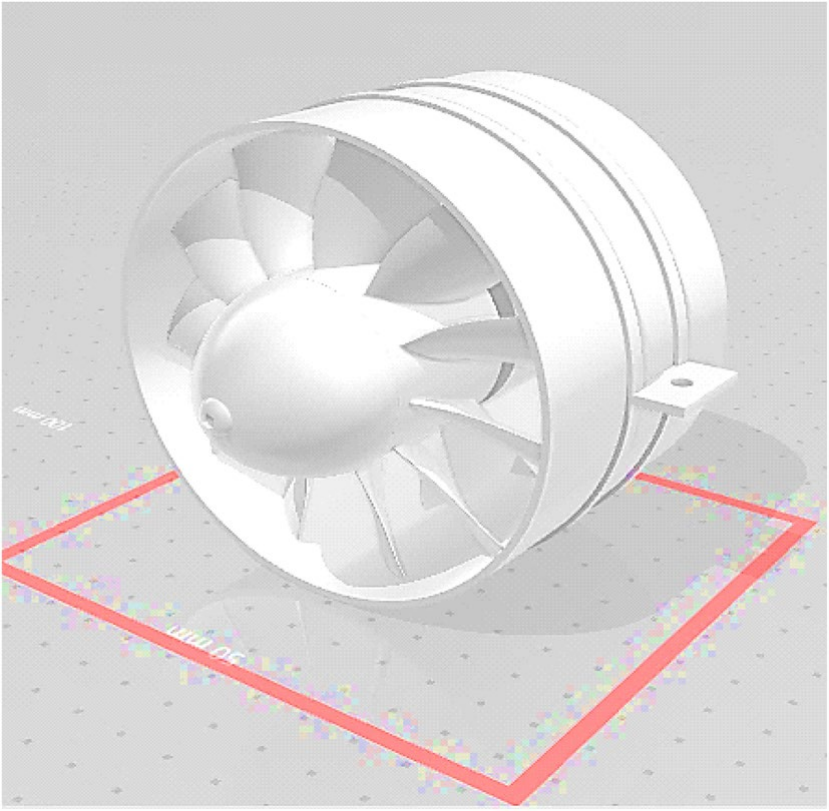
Blade angel 60° + 60° as in Jing et al.^6^.

To check the reliability of the present model, its predictions were compared with those of previous works of Jing et al.^6^, Muthuram^7^, and Anetor et al.^18^. Numerical analysis of the effect of swirl angle and fuel equivalence ratio on the methanol combustion characteristics in a swirl burner reasonable agreement is observed in Fig. 2 that the slight differences between the results may be due to the difference in geometry dimensions. In Fig. 2, a comparison between Jing et al. and our model which have the same boundary condition and the same dimension Jing et al.^6^ we find the same result which is motivated to use the Muthuram^7^ and reach the shown result below where BA2 is blade angel 60° + 60° as in Fig. 3 which is the blade inclination angle butted inside the combustion chamber after the jets as the research Muthuram^7^. The green line in Fig. 2 is our validation and is approximately near the red and blue lines of the Jing et al.^6^ research the simple difference due to the dimension of the combustion chamber.

**Figure 4:**
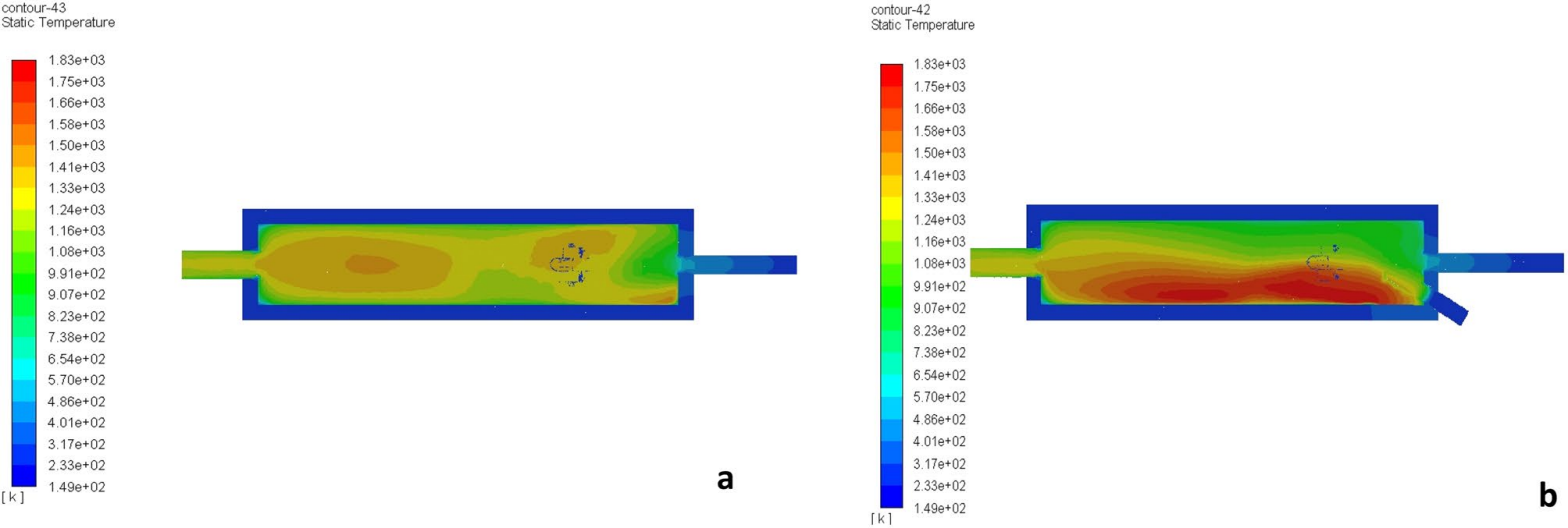
Shape of the flame validation Jing et al.^6^: (**a**) x-axis, (**b**) y-axis.

When the conditions of the search Jing et al.^6^ we gained the same temperature validated to this research a same temp. as shown in Fig. 4. Figure 4 represents the shape of the flame validation in both the x-axis and y-axis according to Jing et al.^6^ model.

## Results and discussions

### Effect of change shapes on combustion honors

The effect of two different shapes (circular, elliptic) which affect the combustion temperature, eff., HHR and equivalent ratio with the variety of different velocities like air or fuels, we divide the result into two parts one with circular and its variation of velocities on the temp., eff., HRR, equivalent ratios, the other is the effect of the elliptic shape of the inlet pipe with velocity variation of air and fuel at the temp., eff. and so on.

### Elliptic shape

#### Effect of change velocities on efficiency, HRR, and equivalent ratios

**Figure 5:**
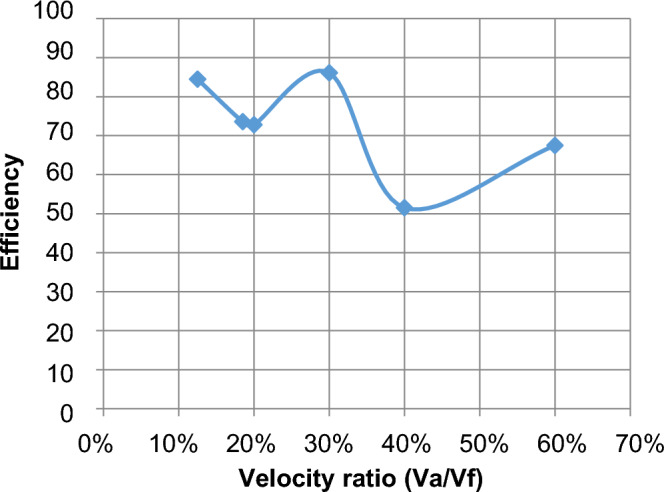
Relation between efficiency as (Y-axis) and percent of (velocity of air/velocity of fuel) as (X-axis).

**Figure 6:**
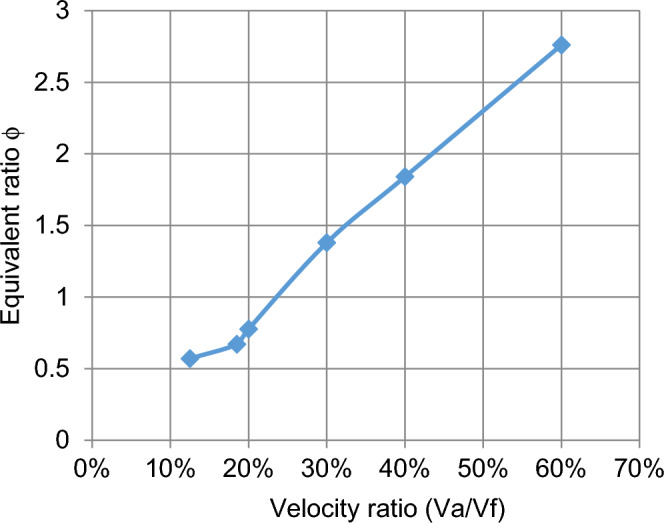
Relation between equivalent ratio and percent of the velocity.

**Figure 7:**
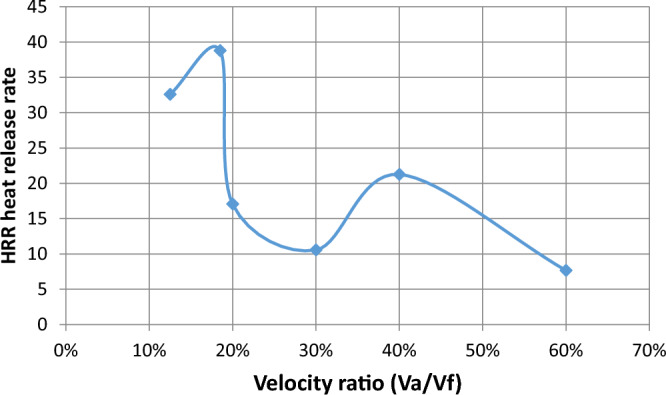
Relation between heat release rate and percent of velocity.

The effect of velocity in the range of study from lean to chemically correct gas mixture on the combustion process is illustrated in Fig. 5 We make a ratio between V_air_ and F_uel_ where we input velocity in air pipe and velocity in two fuel pipes and make a ratio in excel sheet as an X-axis and various in Y-axis of eff., HRR, and so on, We find at percent 13% which represents V_air_ = 12, V_fuel_ = 1.5 and 30% which represents V_air_ = 5, V_fuel_ = 1.5 also affect the eff. To be high then the other values of velocities make it fluctuate until the reaction zone reaches its minimum value then it decreases. Also, the equivalence ratio increases with the increase in the percent velocity of reaction products and this is due to the decrease of the excess air in the fuel/air mixture Fig. 6. Also, the fuel species starts from the inlet boundary value and is kept constant for some distance till the reaction starts then it decreases reaching zero value at the which the reaction stops. Also, the HRR is the maximum value at (v_air_ = 12, vf = 2.22) then the curve is fluctuate at (v_air_ = 2.5, v_fuel_ = 1.5) then increased at percent 40% then slightly decreased this is the effect of variation of velocity to the inlet pipes which connected to the combustion chamber is changed the values of eff. and so on. Figure 7 illustrates the HRR varied values.

#### Effect of inlet methanol, propane, and air temperature

**Figure 8:**
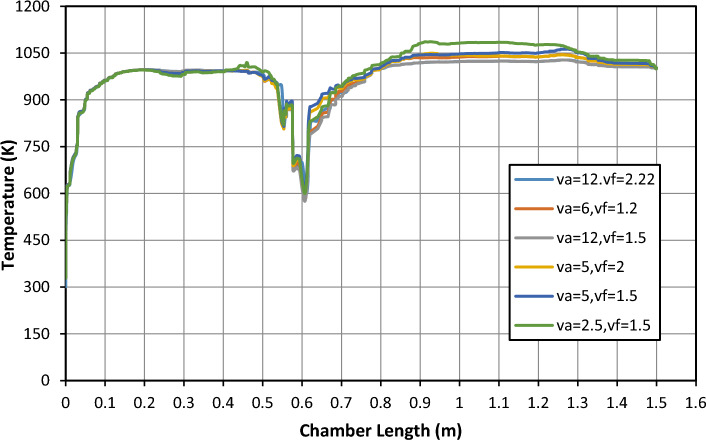
Effect of a velocity change on temperature and combustion chamber length.

**Figure 9:**
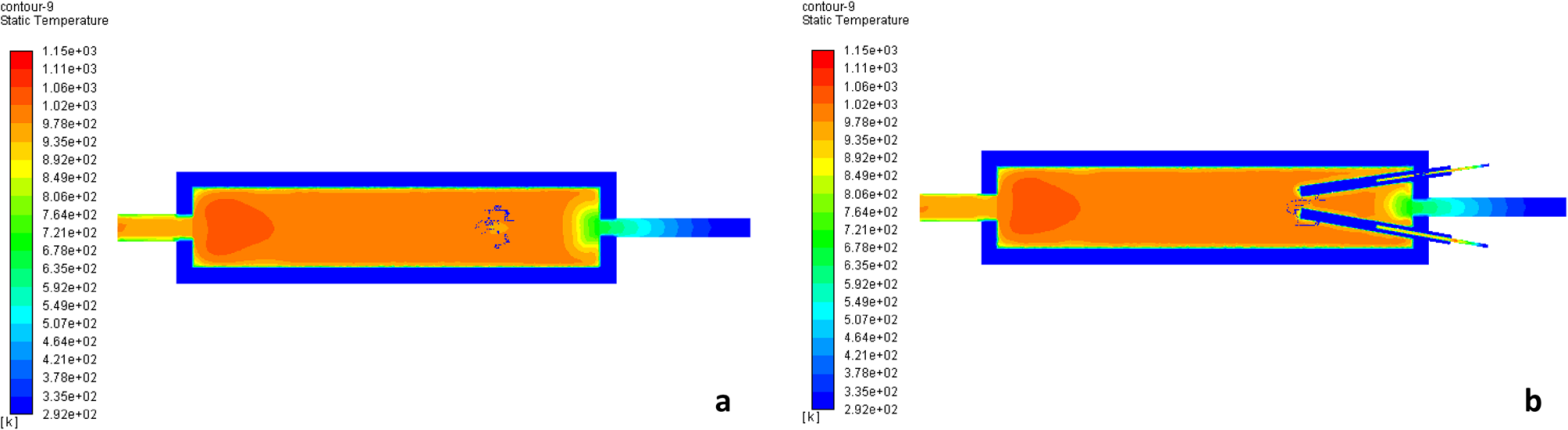
Shape of fire along the center line of the combustion chamber at (**a**) x-axis, (**b**) y-axis.

The inlet effect of methanol, propane, and air temperature on the combustion process is given in Fig. 8. The temperature distribution along the centerline of the combustion chamber is illustrated in Fig. 8. It is shown from the figure that as the inlet temperature decreases, the combustion process starts nearer to the mixture inlet and higher product temperature is attained. The variance velocity is the same temperature it is shown that the curve is increased until the temperature reaches 1100 k as shown, Also the photo in Fig. 9 is the shape of flame in the combustion chamber at V_air_ = 12 and V_fuel_ = 2.22 m/s. This value of velocity has changed the shape of curvature as shown in Figs. 8 and 9.

#### The effect of variation of shape and velocity on combustion behavior

*Circular shape* The change of the shape of pipes at the inlet where one of three inlet pipes is varied like elliptic, or circular changed the result and values of the result as shown.

#### Effect of change velocities on efficiency, HRR, and equivalent ratios

**Figure 10:**
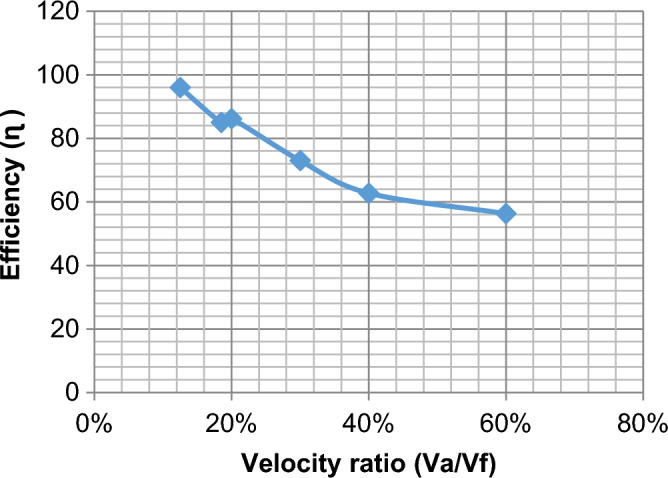
Relation between efficiency as (Y-axis) and percent of (velocity of air/velocity of fuel) as (X-axis).

As shown in Fig. 10 there are extremely different values of efficiency than the elliptic shape, we find variation when variate velocity and shape of the inlet pipes at this Fig. 10 the eff. It decreased slightly from the top point at the percent of velocity 13% which represents (v_air_ = 12, v_fuel_ = 1.5), and then decreased to 56.3% at percent 60% of velocity which represents (v_air_ = 2.5, v_fuel_ = 1.5), by simply explanation we find at Fig. 10 that the efficiency decreased with increasing velocity ratios.

**Figure 11:**
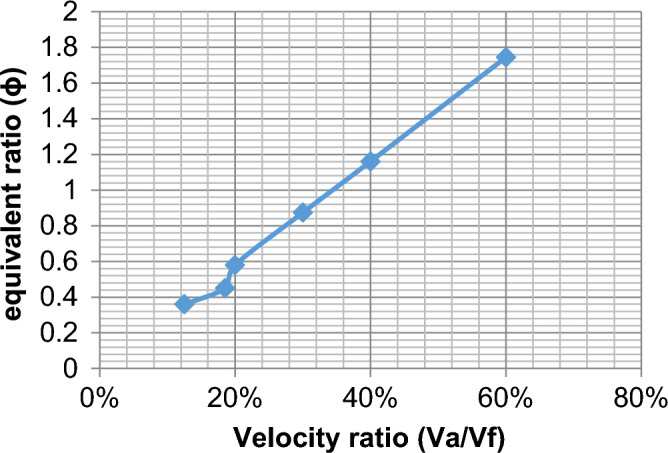
Relation between equivalent ratio as Y-axis and percent of velocity X-axis.

As shown in Fig. 11 the equivalent ratio increased with increasing the velocity ratio and it’s contrasted to efficiency.

**Figure 12:**
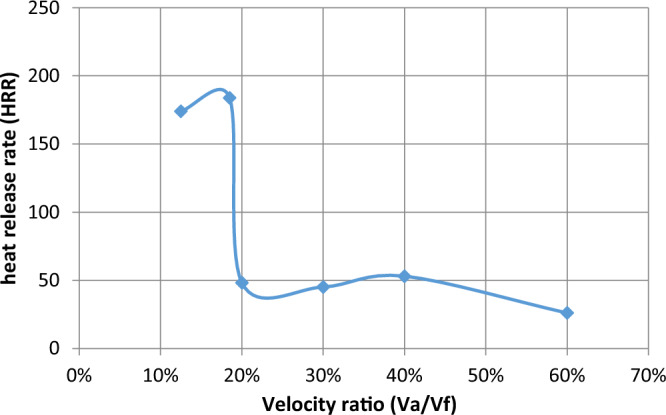
Relation between heat release rate and percent of the velocity.

Figure 12 represents the relation between (heat release rate) and velocity ratio, in this Fig., we find that the heat release rate increased at a velocity ratio of 13% and more increased at 18% then fluctuated after 20% until reached small values as shown. There is enough difference in a curved manner between circular HRR and elliptic HRR but at different velocity ratios it is the same in increasing and then decreasing but the values are different as in Fig. 12 which represents heat release rate as y-axis and velocity ratio in the x-axis where the heat release rate increases with increasing velocity ratio to percent 13% then heat release rate as shown in the figure and different from Fig. 8 for elliptic shape and values is higher than elliptic.

#### Effect of inlet methanol, propane, and air temperature

**Figure 13:**
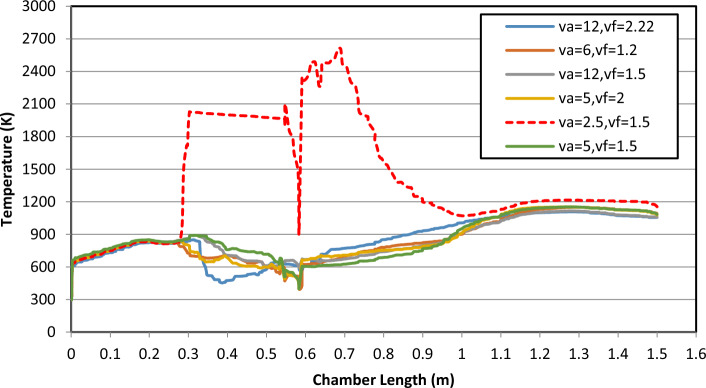
Effect of a velocity change on temperature and combustion chamber length (m).

As shown in Fig. 13 is a relation between the temperature exit from the combustion chamber as y-axis and combustion chamber length as the x-axis and the different color curves is a velocities percent (v_air_/v_fuel_), The effect of changing the velocities and shapes of inlet pipe methanol, propane, on the combustion process is given in Fig. 13 where the temperature distribution along the centerline of the combustion chamber is varied, the temperatures have the same manner for all values of velocity which reach the value 1200 k then decrease as shown but the manner is different at v_air_ = 2.5 and v_fuel_ = 1.5 m/s which represent 40% of air and fuel, we found that temperature is highly increased until reaches 2250 k then decreases as shown in Fig. 13 and have high values of temperature compared to circular–elliptic jet in Fig. 8, so the shape of the inlet jet pipe is affected to the temperature value, and the velocity also in the circular-circular jet is varied the temperature manner which shown in Fig. 13.

**Figure 14:**
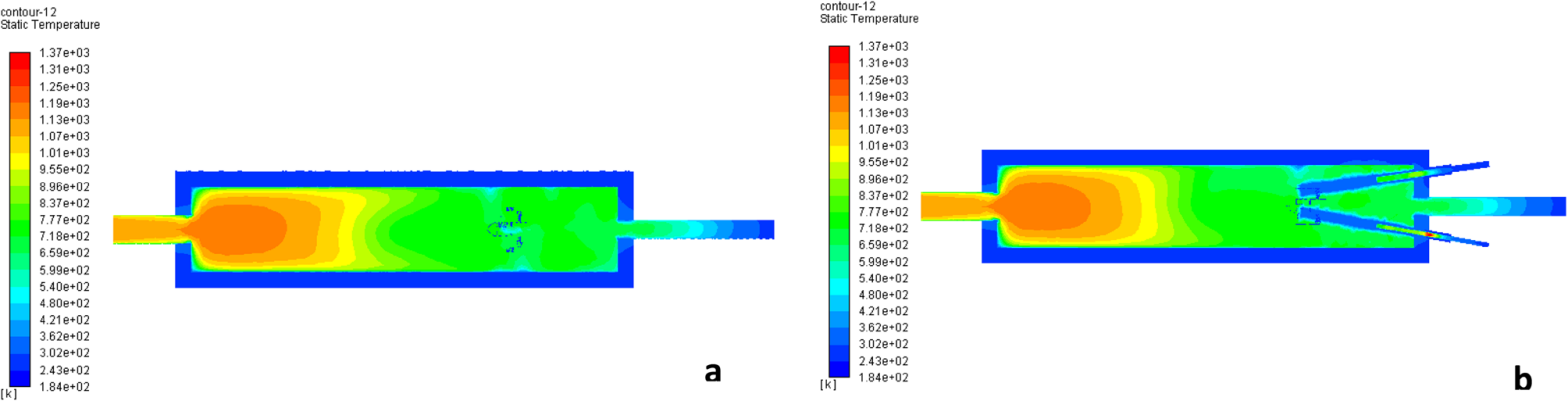
The shape of fire along the center line of the combustion chamber of circular-circular jets (**a**) x-axis, (**b**) y-axis.

Figure 14 represents the shape of fire along the center line of the combustion chamber in the x-axis and y-axis. The figure indicates that the shape of fire inside the combustion chamber which affected by the change of velocity of air and fuel.

### Combustion performance parameters

#### Combustion efficiency

The combustion efficiency is calculated as follows:

The heat liberated from the combustion process is directed to heat both the Q_heat_ to Q_fuel_ plus Q_air_ where Q_fuel_ and Q_air_ represent inputs but Q_heat_ represents the output

9 $${\text{Q}}_{{\text{h}}} = {\text{ m}}_{{\text{g}}} .{\text{ cp}}_{{\text{g}}} \left( {{\text{T}}_{{{\text{go}}}} - {\text{T}}_{{{\text{airi}}}} } \right)$$

10 $${\text{Q}}_{{{\text{air}}}} = {\text{m}}_{{{\text{air}}}} {\text{cp}}_{{{\text{air}}}} \left( {{\text{T}}_{{{\text{out}}}} - {\text{T}}_{{{\text{gi}}}} } \right)$$

11 $${\text{Q}}_{{{\text{input}}}} = {\text{m}}_{{\text{f}}} \times {\text{ l}}_{{{\text{cv}}}} + {\text{Q}}_{{{\text{air}}}}$$

So, the efficiency of the combustion process can be expressed as:

12 $$\eta = {\text{ Q}}_{{\text{h}}} /{\text{Q}}_{{{\text{input}}}}$$

#### Heat release rate

The heat release rate HRR is calculated from:

13 $${\text{HHR }} = {\text{ Q}}_{{{\text{release}}}} /{\text{A}}_{{\text{s}}}$$

where A_s_ is the unit surface area of the combustion chamber

14 $${\text{Q}}_{{{\text{release}}}} = {\text{ Q}}_{{{\text{fuel}}}} + {\text{ Q}}_{{{\text{air}}}} /{2}$$

## Conclusions

From the findings of the current work, there is a difference in the effect of using a circular-circular inlet jet than a circular-elliptic. The effect appeared in efficiency, temperature, equivalent ratio, and HRR. Also, the velocity variance affects the shape and values of the fire. It is observed in the temperature curve for the circular-circular jet that the Air/Fuel velocity ratio of 60% which represents velocity (V_air_ = 2.5, V_fuel_ = 1.5) m/s is the optimum value at which the highest value of temperature than other velocities is obtained so we should use circular-circular jet.

## Data Availability

The datasets used and/or analyzed during the current study are available from the corresponding author upon reasonable request.

## List of symbols

A/F: Air-to-fuel ratio
Cp: Specific heat at constant pressure
Cv: Specific heat at constant volume
D: Mass diffusivity
Da: Damkohler number
E: Energy
Ka: Karlovitz number
k: Turbulent kinetic energy
L: Characteristic length
Le: Lewis number
Lk: Kolmogorov length scale
l0: Integral length scale
m: Mass flow rate
ṁ_a_: Mass flow rate of air
ṁ_f_: Mass flow rate of fuel
P: Pressure
Pr: Prandtl number
L_c_: Length of outer cylindrical
L: Unstrained laminar flame speed
Q: Heat energy
q_w_: Heat transfer to the water heater walls
R: Gas constant
Re: Reynolds number
ReT: Turbulent Reynolds number
Sl: Laminar_flame speed
T: Temperature
Twall: Wall temperature
Tamb: Ambient temperature
TAFT: Adiabatic flame temperature
t: Time
U_f_: RMS velocity_fluctuations
U: Velocity
u: Nusselt number
